# Couple-Level Manifestations of Posttraumatic Stress and Maternal and Paternal Postpartum Relationship Functioning

**DOI:** 10.1155/2024/6140465

**Published:** 2024-03-28

**Authors:** Jordan L. Thomas, Jennifer A. Somers, Christine Dunkel Schetter, Jennifer A. Sumner

**Affiliations:** Department of Psychology, University of California Los Angeles (UCLA), Los Angeles, CA, USA

## Abstract

**Objective:**

Posttraumatic stress disorder (PTSD) is linked with impaired intimate relationships in postpartum women, yet less is known about couple-level manifestations of posttraumatic psychopathology and potential associations with relationship functioning for mothers and fathers during this critical time.

**Method:**

In a predominately low-to-middle income sample of 867 mother-father couple dyads assessed six months following the birth of a child, two analytic methods—a data-driven dyadic latent profile analysis and hypothesis-driven *a priori* categorization approach—evaluated whether discrete subgroups of couples could be identified based on both partners' PTSD symptoms. Structural equation models then tested associations between identified subgroups with (1) self-reported relationship quality and (2) interviewer-rated relationship stress.

**Results:**

Three couple-level PTSD symptom groupings were common to both methods: *both low*, *mother low-father high*, and *mother high-father low*. Dyad-level PTSD symptom patterns were differentially related to relationship dysfunction for mothers and fathers, although mixed findings emerged across methods regarding the relevance of which partner had elevated symptoms for relationship functioning in PTSD symptom-discordant couples. Individuals in dyads characterized by at least one partner with elevated PTSD symptoms consistently exhibited greater relationship dysfunction—indexed both subjectively and objectively—compared to dyads where both partners had low symptoms.

**Conclusions:**

Couple-level typologies of PTSD symptoms can be identified using data- and hypothesis-driven approaches, with generally concordant results. Dyadic patterns of PTSD symptoms are relevant to relationship functioning for both mothers and fathers during the postpartum period and may help to inform more targeted intervention efforts to support couples who are parenting.

## 1. Introduction

Intimate relationships during the perinatal period—the time inclusive of pregnancy and the first postpartum year—have important implications for health. Partner relationship satisfaction declines during the transition to parenthood [1], and psychopathology in one or both partners may strain the couple relationship during this already challenging time. Posttraumatic stress disorder (PTSD)—the sentinel stress-related mental health condition—is increasingly recognized as an important psychological concern with interpersonal consequences, and research has linked maternal PTSD symptom severity with adverse relationship health during the perinatal period (e.g., intimate partner violence [2], poor dyadic adjustment [3], and low relationship satisfaction [4]). However, focusing solely on maternal psychopathology limits understanding of PTSD symptoms in couples and related relationship functioning for both mothers and fathers during this time. Both theory (e.g., couple adaptation to traumatic stress model [5]) and evidence [6, 7] suggest that trauma-related psychological sequelae in one or both partners can impact both individuals in a dyad and the couple system as a whole. Further, trauma exposure—the necessary precursor to PTSD—is common, with nearly 90% of individuals in the general population experiencing at least one traumatic event in their lifetime [8]; thus, the likelihood that both individuals in a couple have experienced trauma is high. Examining dyadic patterns of trauma-related psychopathology may clarify couple-level processes and could inform interventions appropriate for dissemination during the perinatal period.

Two trauma-exposed individuals partnering with one another is well aligned with the phenomenon of assortative mating, or the nonrandom selection of romantic partners based on shared characteristics or experiences. Research has considered assortative mating within married couples based on psychopathology symptom concordance, including in couples with mood [9], anxiety [10], and substance use disorders [11]. Considered in the context of trauma, it may be that both individuals in a couple survived trauma prior to initiating their relationship and that subsequent posttraumatic stress at the dyad-level is compounded by this combination of histories. Known as “dual trauma couples” in the clinical literature [12], this concept has received relatively limited empirical attention, though some research exists. In one population-based study of over 2,000 married couples, exposure to a select traumatic experience (e.g., life-threatening illness/accident, natural disaster, and assault) in one partner was associated with higher likelihood of their partner reporting the same trauma [13]. Importantly, this study was cross-sectional, precluding claims around directionality; however, by definition, childhood maltreatment occurred prior to marriage, thereby lending credence to possible assortative mating based on trauma history.

Findings examining couple similarity of posttraumatic psychopathology—of which trauma exposure is a necessary component—have been more mixed. In one study of Vietnam-era veteran men, those with (versus without) PTSD were more likely to be partnered to women with PTSD [14]. Nearly all (92%) of these relationships were initiated following the veterans' return from deployment, and all women with PTSD reported exposure to trauma outside the relationship, thus providing suggestive evidence for assortative mating. In contrast, another population-based study of older Canadian couples that examined spousal concordance for diverse psychological conditions (*N* = 519 dyads) did not find any couples to be concordant for lifetime PTSD [15]. Importantly, the diagnostic interviews administered in this study utilized *DSM-III*'s diagnostic criteria for PTSD, which differs conceptually from later iterations (e.g., *DSM-IV*, as in Riggs [14]). Moreover, sampling differences across studies—including clinical samples recruited from the Veterans Health Administration versus couples sampled as part of a national population survey—may also contribute to mixed findings, given that only a minority of trauma survivors go on to develop threshold PTSD [8] and that, as a subgroup, veterans exhibit high rates of the diagnosis relative to civilians [16].

Severity of trauma-related psychopathology can also vary between partners, manifesting as either concordance or discordance at the dyad level, and these patterns may have implications for relationship health. Research is mixed regarding whether couples discordant in trauma-related factors—wherein only one partner is considered trauma-exposed or exhibits elevated PTSD symptoms—fare better or worse than their dual trauma/PTSD counterparts. Notably, only a few studies exist in this area, with most in small and circumscribed samples (e.g., military-affiliated and therapy-seeking). Some of this research supports relative equivalence across different couple typologies, wherein relationship satisfaction does not significantly differ between couples where one versus both members have trauma [17, 18]; in other studies, couples with discordant trauma histories—wherein only one partner reports trauma—exhibit better relationship functioning than trauma concordant couples [19]. Though discrepancies across studies may be understood partially as a function of methodological differences (e.g., comprehensive assessments of trauma exposure in Ruhlmann et al. [19] versus more truncated inventories of select events in Nelson and Wampler [17]), qualitative work also supports a range of outcomes in concordant versus discordant couples. One interview study of 22 single and dual trauma couples found that while both groups attributed some relationship strengths to having experienced trauma (e.g., support), some dual trauma couples endorsed posttrauma relationship difficulties (e.g., communication problems related to trauma and trauma triggers in the context of the relationship [20]). Thus, different dyads may be differentially affected by similarity in trauma-related factors.

One analytic method through which to examine concordance versus discordance of trauma-related psychopathology in couples is through latent classification methods such as latent profile analysis (LPA). While less commonly applied to dyadic data, these approaches focus on empirically identifying homogenous subgroups within the broader population, and growing research has considered such methods in the context of couples. By including indicators drawn from each partner, profiles representing discrete subgroups of couples based on these constructs are formed. To date, only one latent classification study has aimed at elucidating couple categorizations based on both partners' mental health symptoms. This research, conducted in a sample of different-gender couples, found evidence for four profiles, including two characterized by symptom concordance (e.g., both partners high in symptoms of depression and anxiety and both partners low in symptoms) and the remaining characterized by discordance (e.g., man higher in symptoms and woman higher in symptoms [21]). To our knowledge, only one study has utilized a latent classification approach to specifically examine PTSD symptoms within dyads, and this study was conducted in a sample of violence-exposed women and their children [22]. Using LPA, the authors found two profiles based on both informants' PTSD symptoms, with mothers and their offspring in each profile reporting similar scores to one another—or relative symptom concordance—in each subgroup. A natural next step in this work is to examine functional correlates of these types of dyadic categorizations.

To date, only two studies have examined PTSD and relationship functioning in couples during the perinatal period, with somewhat contradictory findings. One, a cross-sectional study of 64 couples assessed 9-week postpartum, only measured intrusion and avoidance PTSD symptoms; these symptoms were significantly correlated within couples (*r*s = .37–50), highlighting a trend toward concordance in the broader sample. However, neither maternal nor paternal PTSD symptoms were linked with either partner's self-reported relationship satisfaction [23]. Although these results should be interpreted cautiously given methodological concerns (e.g., small convenience sample, limited measure of PTSD symptoms, and no statistical adjustment for within-couple interdependence), it may be that partners' concordance in these specific PTSD symptom clusters served a protective function for relationship health, which could explain why no associations were detected. In the other study, which sampled 250 predominantly married couples 10 months after birth, maternal and paternal total PTSD symptoms were also correlated within couples (*r* = .23), again suggesting relative concordance in the sample; both partners' PTSD symptoms were negatively linked with their own and their partner's couple functioning [24]. However, in both of these studies, the independent variables were structured at the individual level (e.g., maternal PTSD symptoms and paternal PTSD symptoms) rather than at the couple level; thus, it is unknown whether associations may have differed according to whether partners were concordant or discordant in PTSD symptoms.

In this study, we investigated couple-level manifestations of PTSD symptoms and associations with relationship functioning in a sample of mother-father couple dyads during the postpartum period. First, we assessed if distinct subgroups of couples—based on patterns of both partners' total PTSD symptoms—could be identified. We simultaneously leveraged two analytic methods—a data-driven and a hypothesis-driven approach—to explore this. For the data-driven approach, we employed dyadic LPA to test whether couples could be statistically grouped based on combinations of both partners' PTSD symptoms. Although LPA is hypothesis-free, we anticipated certain, theoretically relevant subgroups to emerge; specifically, we predicted couples where both partners exhibited elevated PTSD symptoms (*both high*); couples where neither partner exhibited elevated symptoms (*both low*); couples where only the mother exhibited elevated symptoms (*mother high-father low*); and couples where only the father exhibited elevated symptoms (*mother low-father high*). Given limited prior work, we also explicitly modeled these subgroups as *a priori* categorizations in a hypothesis-driven approach and explored overlap between these two classification methods.

Second, we investigated associations between these couple-level categorizations of PTSD symptoms and subjective and objective indices of relationship functioning for each partner. In prior work in a subset of the women in this cohort, mothers with higher total PTSD symptoms exhibited greater interviewer-rated stress in their partner relationship and reported lower relationship quality [25]. We hypothesized that couples where one or both partners demonstrate elevated PTSD symptoms (*both high*, *mother low-father high*, and *mother high-father low*) would have poorer relationship functioning relative to couples where neither partner exhibits elevated symptoms (*both low*)—with largest effects observed for PTSD concordant couples (*both high*). We also evaluated whether these couple-level PTSD symptom manifestations would be more strongly linked with relationship functioning for mothers versus fathers. Given minimal evidence in this area, we did not predict gender-based divergences.

## 2. Methods

### 2.1. Participants and Procedure

Data were from the Community Child Health Network (CCHN), a community-academic partnership dedicated to the investigation of maternal and child health disparities. Using community-based participatory research methods, CCHN conducted a five-year, prospective study of newly postpartum women and the fathers of their children [26]. Black, Latina, and White mothers were recruited at delivery of a child in one of five U.S. sites, including three urban areas (Los Angeles, CA; Baltimore, MD; and Washington, DC), one suburban location (Lake County, IL) and one rural site (counties in eastern North Carolina); those interested in participating in CCHN provided consent, and fathers were invited to participate with the consent of mothers. Both mothers and fathers were assessed individually at one-month postpartum and then every six months for two years via in-home interviews. Community members trained in methods and academic research staff trained in community research conducted the in-home interviews in either English or Spanish. Protocols were approved by the Institutional Review Boards of all study partners. Additional information on CCHN is described elsewhere [26, 27].

### 2.2. Transparency and Openness

For this study, we report how we determined sample size, all data exclusions, and all measures. We leveraged maternal and paternal data that was collected six month postpartum, the only timepoint at which PTSD symptoms were assessed in CCHN. The analytic sample comprised the 867 couples in which both partners had complete PTSD data. This study was not preregistered; materials (e.g., data and code) are available from the first author upon reasonable request.

### 2.3. Measures

#### 2.3.1. PTSD Symptoms

At six months postpartum, PTSD symptoms were assessed using the PTSD Checklist-Civilian Version (PCL-C) [28]. Both partners independently indicated how bothered they were by each of the 17 *DSM-IV* PTSD symptoms in the past month, with responses rated on a 5-point scale (1 = not at all, 5 = extremely) and summed to create a total symptom severity score (possible range 17-85; Cronbach's *α* = .91 for mothers, *α* = .92 for fathers). A well-validated and reliable measure [29], the PCL has been used to assess PTSD symptoms in perinatal populations (e.g., [25]). We considered total scores ≥ 30, a cut-off that was initially validated in an HMO sample of community-dwelling women [30], as representative of potential maternal PTSD based on elevated symptoms. We considered scores ≥ 31 to indicate elevated paternal PTSD symptoms, a score identified in a sample predominately comprised of veteran men [31]. These cut-offs were used for descriptive purposes and for the *a priori* hypothesis-driven categorization approach.

As in most prior research using this measure(for a review, see McDonald and Calhoun [32]), the PCL in this study was not anchored to a specified index trauma; rather, participants reported symptoms as anchored to “general stressful experiences.” This adaptation was made so as to capture PTSD symptomatology due to multiple possible sources—including non-Criterion A stressors such as discrimination or other adverse experiences—in this predominately low-income population. However, in service of ensuring these symptoms were truly trauma-related, we also collated available data from another measure in CCHN—the Life Events Checklist [33]—to determine potential Criterion A trauma exposures predating the PTSD symptom measure. One month following delivery, mothers and fathers independently reported on life events and indicated whether they or close others had experienced each event over the past year. Consistent with the definition of a *DSM-IV* Criterion A trauma (e.g., individual directly experienced or witnessed event(s) involving actual or threatened death, significant injury, or threat to physical integrity [34]), we selected eight events from the checklist as potentially traumatic experiences (serious injury, illness, hospitalization; mugging or assault; death; vehicular accident; threat of physical harm by another person; robbery/burglary; natural disaster; and victim of violent crime) and summed these to create a past-year trauma burden score for both mothers and fathers. As cumulative trauma exposure is linked with greater PTSD symptom severity [35], we then examined correlations between these past-year trauma burden scores and individuals' total PTSD symptoms, as done in prior CCHN research on maternal PTSD [25].

#### 2.3.2. Relationship Functioning

Relationship functioning at six months postpartum was indexed by two separate measures: self-reported *relationship quality* and objectively indexed *partner relationship stress.* Each assessment was completed independently by mothers and fathers in separate locations.

Partners separately reported their *relationship quality* using the 32-item Dyadic Adjustment Scale (DAS) [36], which is comprised of four subscales: Dyadic Consensus (the degree to which couples agree on topics, e.g., career decisions and household tasks), Dyadic Satisfaction (the degree to which couple is satisfied with their relationship), Dyadic Cohesion (the degree of closeness and shared activities within the couple), and Affective Expression (the degree to which couples demonstrate physical affection for one another). Item-level responses are summed to create a total scale score (possible range 0-151), where higher scores indicate more positive relationship adjustment, alternatively referred to as relationship quality. Internal consistency was high for both *maternal relationship quality* (*n* = 688, *α* = .93) and *paternal relationship quality* (*n* = 728, *α* = .92). A psychometrically sound measure [37], the DAS has been shown in factor analytic work to be a gender-invariant assessment of total relationship adjustment [38].

Both partners also independently completed an adapted version of the UCLA Life Stress Interview (LSI), a semistructured interview of chronic stress in various life domains [39]; this resulted in a *partner relationship stress* indicator for mothers (*n* = 713) and a *partner relationship stress* indicator for fathers (*n* = 776). Modifications to the gold standard LSI were done in conjunction with CCHN community partners and interviewers and piloted prior to implementation. Measure adaptations included shortening the overall interview, simplifying interviewer instructions, and adding and amending questions (e.g., using term “relationship partner” rather than “spouse”). As part of the *partner relationship stress* domain, both mothers and fathers reported on features of their intimate relationship over the past six months, including commitment and stability; closeness, trust, and confidence; support and dependability; and conflict resolution. Highly trained assessors assigned objective stress severity ratings using a 5-point Likert scale with behaviorally specified anchor points (1 = exceptional relationship that is close and trusting, long-standing, and stable, with good conflict resolution; 5 = highly negative relationship that is unstable and uncertain, lacks communication and trust, and is physically or emotionally abusive), where higher scores indicated greater stress. Each response was coded by one interviewer. A subset of responses from mothers in the broader CCHN sample were analyzed as part of a reliability analysis; intraclass correlations estimating interrater reliability ranged from .64 to .76, indicative of substantial reliability [40]. For more information on the administration and scoring of the LSI in CCHN, see Tanner Stapleton et al. [40].

#### 2.3.3. Sociodemographic and Relationship Characteristics

Both mothers and fathers self-reported sociodemographic information at enrollment, including age, racial and ethnic identity (with mothers categorized as Latina, Black, and non-Hispanic White due to recruitment method; fathers were grouped into the same categories, with an additional “other” category inclusive of individuals identifying as Asian, American Indian, multiracial, and other races not identified), highest level of education attained, and income. Mothers also reported parity.

Due to research substantiating couple similarity in sociodemographic factors [41], we created dyad-level variables to capture couple-level characteristics for descriptive purposes and, when pertinent, to model as covariates in the structural analyses, as the couple dyad was the primary unit of focus. These variables included *age*, which involved averaging the age of both partners; *minority racial and ethnic couple status*, a binary variable indicating couples where both partners self-identified as persons of color; and *dyad-level education* and *dyad-level poverty*, which, respectively, captured the highest level of education attained and highest income level across both members of the couple. We also included a *committed relationship* indicator, which combined marital (married versus not) and cohabitation (living together versus not) status into a single variable, given that couples in this study were united by the birth of a baby rather than solely by marriage.

### 2.4. Analytic Plan

First, we examined descriptive statistics to characterize our sample. We then conducted within-couple correlations on the primary study variables of PTSD symptoms and relationship functioning. To add credence to the PTSD symptom assessment, we leveraged available data on past-year trauma exposure to perform a sensitivity analysis, wherein we examined correlations between a count measure of recent trauma exposure in mothers and fathers and their total reported PTSD symptoms.

Second, using Mplus version 8 [42], we conducted dyadic LPA using mothers' and fathers' total PTSD symptom scores as the two continuous indicators. To determine the optimal number of profiles, we compared model fit values across conventional model fit statistics: log likelihood, Akaike information criterion (AIC), Bayesian information criterion (BIC), and sample-size adjusted BIC (SABIC). Across these indicators, lower values generally indicate more optimal fit [43]. Two maximum likelihood tests—the Lo-Mendell-Rubin likelihood ratio test (LMRT) and the bootstrap likelihood ratio test (BLRT)—then tested whether systematic addition of subsequent profiles improved overall model fit. Entropy, which measures the distinguishability of profiles generated by each LPA model, was also consulted; plausible values for this indicator range from 0 to 1, where higher numbers reflect higher classification accuracy. We also considered model interpretability, in the form of theoretical relevance and relative size of the emergent typologies (e.g., profiles containing at least 5% or more of the sample [43]). For interested readers, we present the average PTSD symptom scores for mothers and fathers in each profile among the different profile solutions in Supplementary Table 1. When to include covariates is an ongoing area of discussion in the latent classification literature, as incorporating additional variables into models may alter resultant typologies in theoretically inconsistent ways, and simulation studies support first determining the optimal model prior to considering covariates [44]. For this reason, we elected to focus on PTSD symptoms as the sole indicators in the LPAs and to include pertinent sociodemographic covariates in the structural analyses.

In addition to identifying couple profiles through LPA, we grouped dyads based on *a priori* cut-offs (e.g., both partners exceed score indicative of elevated PTSD symptoms, neither exceeds, and one exceeds) to explore whether these hypothesis-driven groupings mapped on to the LPA profiles. As a latent classification method, LPA will always identify subgroups in a dataset, regardless of theoretical relevance of the selected indicator variables. Thus, we aimed to complement this data-driven approach with a theory-driven classification method that capitalized on existing cut-off scores. We then evaluated the degree of overlap between approaches—that is, considering whether dyads were classified identically across both the data- and hypothesis-driven categorization methods—in an exploratory comparison.

Third, using the profile membership classifications and the *a priori* categorizations (e.g., both partners exceed elevated PTSD symptoms cut-off, neither exceeds) as predictors, we examined associations between these and the self-reported and interviewer-rated relationship functioning of both mothers and fathers through a series of structural equation models (SEMs) in Mplus. As a multivariate technique, SEM can include both latent and manifest variables, as well as adjust for covariance between variables in the event of interdependence. All mothers and fathers in the analytic sample had complete PTSD symptom data; however, missingness on the relationship functioning measures ranged from 10.50% (partner relationship stress indicator for fathers) to 20.65% (maternal relationship quality). In addition, maternal and paternal PTSD symptoms were correlates of missingness on maternal relationship functioning and paternal relationship functioning, respectively. To address this, we integrated full information maximum likelihood (FIML) estimation with cluster robust standard errors (MLR); FIML produces unbiased maximum likelihood estimations under missing at random conditions, while MLR adjusts for the nonindependence of dyadic data.

In these structural models, both sets of independent variables—the profile membership and *a priori* groupings—were coded as dummy variables, with the largest category considered as the reference group in analyses examining links between the dyadic classifications and relationship functioning. We considered different reference groups in a series of supplemental analyses (Supplementary Table 2 uses LPA-derived memberships and Supplementary Table 3 uses the *a priori* groupings) to test whether other subgroups meaningfully differed from one another.

The primary analyses involved two sets of models that varied based on informant reporting method of the relationship outcome (e.g., self-report versus interviewer-rated). That is, the *partner relationship quality* and *partner relationship stress* measures were analyzed separately. To account for partner interdependence within each of these relational constructs, we used the WITH command in Mplus to generate unbiased estimates of the standard errors [45]. To adjust for interdependence of additional within-couple features, we included a series of dyad-level covariates in all structural models if these factors were significantly linked with maternal and paternal total PTSD symptoms. These covariates included dyad-level age, poverty, and education, as well as the committed relationship indicator.

## 3. Results

### 3.1. Descriptive Statistics for the Overall Sample

The analytic sample included 867 mother-father couple dyads (Table 1). On average, the individuals in these couples were in their late twenties; the majority (75.5%) were in a committed relationship. Two-thirds of dyads (67.8%) were comprised of individuals who both held minoritized racial and ethnic identities, with Black being the most represented race. When examining the highest level of education attained at the dyad level, the largest proportion of couples (37.9%) held high school degrees. Income was variable; while one-half of dyads reported living > 200% above the federal poverty line, the remaining lived below this threshold.

### 3.2. Bivariate Relationships among Primary Study Variables

Within-couple correlations among the primary study variables—PTSD symptoms and the two indicators of relationship functioning—were all significant. Maternal and paternal total PTSD symptoms were positively correlated with one another; the magnitude of the association was small to moderate (*r* = .25). Self-reported relationship quality (*r* = .56) and interviewer-rated relationship stress (*r* = .68) were correlated with large effect sizes within couples.

### 3.3. Trauma Exposure Sensitivity Analysis

As seen in Table 1, among fathers with available data on both past-year trauma and current PTSD symptoms (*n* = 684), greater trauma burden was significantly correlated with more paternal total PTSD symptoms (*r* = .27). A significant association was also observed between mothers' past-year trauma burden and total maternal PTSD symptoms, though the effect size of this correlation was smaller in magnitude (*n* = 839, *r* = .11).

### 3.4. LPA to Identify Couple Subgroups

In order to determine the optimal number of latent profiles, we examined one, two, three, four, and five profile solutions. As the number of profiles increased, the log likelihood, AIC, BIC, and sample-sized adjusted BIC values decreased, indicating improved model fit. Entropy—an indicator of classification accuracy—was high across solutions and generally improved as the number of profiles increased (see Table 2 for model fit indices). The one exception to this was with the five-profile solution, which yielded lower entropy. Maximum likelihood tests revealed that across the one, two, three, and four class solutions, each increasingly complex model provided significantly better fit than its predecessor. These findings were consistent across the two likelihood ratio test statistics, LMRT and BLRT; however, results regarding the five-profile solution were mixed across these indicators, with the LMRT suggesting poorer fit relative to the four-profile model and the BLRT indicating more improved fit (Table 2).

We also compared the theoretical relevance of the generated PTSD means for the two, three, four, and five profile solutions (Supplementary Table 1). The four-profile solution, which emerged as the most favorable result based on model fit statistics and maximum likelihood tests, was also the most theoretically consistent, in that it was comprised of two profiles characterized by couple concordance in PTSD symptoms (both low, both high) and two symptom discordant profiles (profile where mothers are high, fathers are low; profile where mothers are low, fathers are high). However, the profile comprised of dyads where both partners reported elevated PTSD symptoms constituted only 1.3% of the sample and thus did not reach the recommended profile size threshold. Based on this, a three-profile solution was selected as most optimal. The emergent profiles from this solution included one symptom concordant profile—wherein both partners in the couple reported minimal PTSD symptoms—and two symptom discordant profiles, where one partner reported substantially more PTSD symptoms than the other. We assigned labels to the profiles that captured this, as follows: (1) *both low*, (2) *mother low-father high*, and (3) *mother high-father low*. These profiles, respectively, comprised 81%, 7%, and 12% of the sample dyads.

### 3.5. Associations between LPA-Derived Couple Profiles and Relationship Functioning

Models examining associations between the LPA-identified profiles and relationship functioning metrics—adjusting for dyad-level sociodemographic correlates and the relationship commitment indicator—were significant, accounting for between 12 and 18% of the variance in relationship health for mothers and fathers (Table 3). Associations were of similar magnitudes across partners and metrics and, overall, reflected worse relational functioning among couples discordant in PTSD symptoms relative to couples where both partners reported minimal symptomatology. More specifically, relative to mothers in *both low* couples, mothers in the *mother high-father low* profile demonstrated significantly lower relationship quality (*β* = −0.70) and greater relationship stress (*β* = 0.77); the same pattern held for fathers in this profile (relationship quality: *β* = −0.57, relationship stress: *β* = 0.64). Likewise, compared to individuals in the *both low* couples, mothers and fathers in the *mother low-father high* subgroup also exhibited more adverse relationship quality (maternal *β* = −0.61, paternal *β* = −0.61) and stress (maternal *β* = 0.77, paternal *β* = 0.71). Though there were gender differences in the magnitude of the effect sizes, confidence intervals were overlapping, suggesting generally consistent findings for mothers and fathers. Analyses considering different reference groups indicated no significant differences between mothers and fathers in the symptom discordant subgroups across either of the relationship functioning indicators (Supplementary Table 2).

### 3.6. A Priori Couple Classifications

As part of a hypothesis-driven approach, couples were then classified using cut-off scores suggested by the literature to be approximates of clinically significant PTSD symptoms. Similar to the LPA-derived classifications, most dyads (62.5%) were captured by a *both low* group. As was the case with the latent modeling approaches, there were also two categorizations representing symptom discordance based on gender—that is, a *mother low-father high* group (13.3%) and a *mother high-father low* group (15.6%). However, unlike the LPA profiles, the *a priori* categorizations included a group of *both high* couples (8.7%), wherein both partners reported high levels of total PTSD symptoms—representing another form of symptom concordance.

### 3.7. Exploratory Comparison of Data- and Hypothesis-Driven Couple Categorizations

Approximately three-quarters of the dyads (73.5%) were categorized identically across both the LPA and *a priori* classification methods. Of those remaining, a small proportion (8.7%) were categorized as *both high* in the hypothesis-driven approach and as *both low* (*n* = 9), *mother low-father high* (*n* = 19), and *mother high-father low* (*n* = 47) in the LPAs. The remaining dyads (17.9%) were primarily coded by the data-driven approach as *both low* and by the *a priori* methods as either *mother high-father low* (*n* = 82) or *mother low-father high* (*n* = 73).

### 3.8. Associations between A Priori Couple Profiles and Relationship Functioning

Table 4 details associations between the *a priori* categorizations and relationship metrics; a similar amount of variance was accounted for by these models (14-19%) as those that utilized the LPA profiles. As was the case with the LPA-derived groups, relative to their counterparts in the *both low* dyads, mothers and fathers across all other symptom manifestations—including the *both high* couples—exhibited significantly greater relationship stress and lower relationship quality, even when controlling for pertinent covariates. Robust differences by gender were again not observed, as evidenced by overlapping confidence intervals. The one exception to this general pattern is that the differences in reported relationship quality between mothers in the *both low* and *mother low-father high* groups did not reach statistical significance.

Analyses considering alternative reference groups revealed slightly different results than those obtained using the LPA-identified classifications. Notably, using these hypothesis-driven categorizations, significant differences were observed between the two symptom discordant groups with regard to relationship functioning (Supplementary Table 3). Specifically, partners in discordant couples reported greater relationship quality when they were the one with low, rather than high, PTSD symptoms; this was observed for both mothers and fathers. The same pattern was observed for mothers, but not for fathers, with the relationship stress indicator. Lastly, individuals in the *both high* dyads exhibited lower relationship functioning relative to their counterparts in some, but not all, symptom discordant groups; this effect was consistent across gender and appeared to vary as a function of whomever in the dyad reported higher PTSD symptoms, with partners exhibiting better relationship functioning compared to those in *both high* couples when they were the one with low symptoms in the discordant couple. More specifically, mothers in the *mother low-father high* dyads and fathers in the *mother high-father low* couples demonstrated more optimal relationship functioning than individuals in the *both high* couples; the other comparisons revealed no significant differences.

## 4. Discussion

Posttraumatic psychopathology is linked with intimate relationship difficulties [6, 7], including during the perinatal period (e.g., [25]), but most research on these associations adopts an individual-level approach by considering only one partner as trauma-exposed. Examining PTSD in couples—both through assessing trauma-related psychopathology across partners and by considering dyadic patterns—could inform treatments to improve relationship functioning and how best to allocate those interventions. In this study, we explored couple-level manifestations of PTSD symptoms at a critical time: the postpartum period. Utilizing data- and hypothesis-driven approaches and the largest sample of postpartum couple dyads studied to date, we examined if distinct subgroups of dyads could be identified based on both partners' PTSD symptoms. Across methods, we found evidence supporting discrete subgroups, including couples characterized by concordance in minimal PTSD symptoms (*both low*) and symptom discordance (*mother low-father high*, *mother high-father low*). We also examined associations between the couple-level categorizations and indicators of relationship functioning for mothers and fathers. Evidence for differential associations emerged; overall, within each categorization approach, results were relatively consistent for mothers and fathers and across the informant reporting methods.

Classifying couples according to both partners' PTSD symptoms revealed that a range of different subgroups existed in this large, diverse community-based sample. Relative consistency was observed between the couple-level classifications elucidated by the latent classification and the theory-based groupings imposed on the data. Indeed, nearly three-quarters of the sample were categorized identically across approaches. These classifications—*both low*, *mother low-father high*, and *mother high-father low*—even captured the majority of the sample in the *a priori* approach, highlighting the value of latent classifications. The remaining couples in the *a priori* categorization represented dyads where partners were *both high* in symptoms, a subgroup that was identified by a four-profile solution in the LPAs. However, only 1.3% of the dyads comprised the *both high* category in this result, rendering a three-profile solution as preferable. Given that these couples were unselected for trauma exposure or PTSD, it is likely that the skew of PTSD symptoms limited capacity to empirically parse as many dyads into a *both high* classification, instead relegating them to more homogenous subgroups of symptom discordance.

Nevertheless, simultaneously implementing data- and theory-driven approaches to classify couples according to both partners' PTSD symptoms can help clarify dyadic patterns, as each method has its own advantages and limitations. As an empirically based approach, latent classification methods can uncover previously undetected subgroups; at the same time, these data-driven methods will always identify categories, regardless of their theoretical relevance. While hypothesis-driven approaches directly rely on theoretical precedent to form subgroups, the scores that shaped the categorizations in this study were originally validated in homogenous, circumscribed samples (e.g., healthcare-seeking sample of veteran men in Yeager et al. [31]) and thus potentially are not as sensitive to variation in more diverse populations. Used in conjunction, these approaches validate the existence of different subgroups of dyads based on both partners' PTSD. The only other latent classification study to model PTSD symptoms in dyads focused on mothers and children, and the generated profiles largely reflected symptom concordance [22]. In contrast, here, we empirically identified profiles characterized by symptom discordance, as well as one subgroup of symptom concordance. This latter group captured couples concordant in minimal PTSD symptoms, similar to dyads concordant in “no psychopathology” as in prior research [11]. The empirical categories that emerged here also mirror those generated in a latent classification study of psychopathology symptoms in therapy-seeking couples (e.g., partners concordant in low symptoms and partners discordant in symptoms based on gender; [21]), as well as those in a cluster analysis that identified subgroups based on both partners' childhood trauma exposure (e.g., both low, medium—high, and high—medium; [18]). While no subgroup of dyads concordant in high PTSD symptoms emerged in the empirical approach, this theory-driven grouping reflects categorizations observed in other psychopathology concordance studies and embodies the principle of assortative mating.

Across the two classification methods, the most consistent finding was that relative to their counterparts in the *both low* dyads, mothers and fathers in couples with at least one partner with elevated PTSD symptoms exhibited greater interviewer-rated relationship stress. Similar patterns were observed with regard to the self-report indicator of relationship quality, again highlighting that both mothers and fathers in couples characterized by PTSD displayed less optimal relationship functioning than their counterparts with minimal symptoms. Some important differences across methods were also observed. Using the LPA-derived groupings, couples discordant in PTSD symptomatology—that is, the *mother high-father low* and *mother low-father high* dyads—did not differ from one another with regard to relationship quality or stress. In other words, the gender of the partner with elevated PTSD symptoms was not differentially associated with the relationship functioning measures in mothers or fathers. This was not the case with the *a priori* categorizations, wherein there was graded discrimination between these two subgroups across both relationship indicators and partners. Indeed, using the theory-driven groupings, individuals who were low in symptoms when their partners were high (e.g., mothers in the *mother low-father high* dyads) exhibited more optimal relationship functioning than their counterparts in the other symptom discordant group (e.g., mothers in the *mother high-father low* group). This pattern held for both mothers and fathers and was consistent across informant methods, with one exception: fathers in the *mother low-father high* dyads did not differ from those in the *mother high-father low* subgroup on relationship stress.

Though the *both high* dyads emerged only in the theory-driven approach, comparisons between this subgroup and the others yielded interesting findings worthy of mention. Indeed, relative to their counterparts in the *both high* dyads, neither mothers nor fathers in symptom discordant couples benefitted from having a partner low in symptoms. That is, mothers in the *mother high-father low* and fathers in the *mother low-father high* groups exhibited similar relationship functioning as their counterparts in the *both high* couples; this was consistent across the subjective and objective relationship indicators. It may be that in the presence of any PTSD in a dyad, the relationship health of individuals is more strongly influenced by their own, rather than their partner's, symptoms. These individuals' high levels of PTSD symptoms may then prevent them from engaging with their partners, who would likely be less affected by pathology.

Results complement and extend prior work considering trauma and PTSD in couples. For instance, the finding that couples characterized by any elevated PTSD symptoms—whether endorsed by one or by both partners—experience lower relationship functioning than couples where both partners are low in symptoms is consistent with previous research considering single trauma, dual trauma, and non-trauma-exposed couples [17]. Moreover, the finding that both partners' PTSD symptoms are relevant to relationship functioning is consistent with some (though not at all; [23]) prior work conducted in couples during the postpartum period [24]. However, it is important to note that the current study utilized a dyad-centered approach—where the predictor represented couple-level patterning of PTSD—rather than the individual-level perspective adopted by prior work, in which both partners' PTSD symptoms are considered as individual predictors. These different approaches—where the level of analysis varies—complicate direct comparisons of findings.

While we cannot directly test why individuals partnered with others either concordant or discordant from them in PTSD symptoms, we offer some hypotheses. Most couples here were characterized by both individuals reporting minimal PTSD symptoms; importantly, the descriptor minimal is relative, given that the average levels of symptomatology in these couples were nonzero. Nevertheless, the identification *of both low* dyads is consistent with concordance of “nonpsychopathology” in other studies of community-based couples [11], as well as likely reflects low base rates of elevated PTSD symptoms in the general population. Indeed, although trauma exposure is common, most individuals do not develop PTSD [8]; thus, concordance in minimal symptomatology is expected, particularly in non-clinical samples. Moreover, aligned with assortative mating, trauma-exposed individuals may have been drawn to other survivors based on similar PTSD manifestations, even if both partners' symptoms were relatively minimal. Indeed, in our dyads, symptoms were significantly correlated within couples, with small-to-moderate effect sizes, as comparable to estimates from other work on couple similarity in psychopathology [41]. Undoubtedly, individuals mate for a variety of both genetically (e.g., height) and socially determined (e.g., religion) traits beyond PTSD symptoms or the vulnerabilities to negative affect that confer psychopathology risk; thus, couple similarity in unassessed third variables may also explain these groupings.

Irrespective of how these individuals partnered, several mechanisms may explain why relationship functioning varies by couple-level PTSD. The cognitive, emotional, and interpersonal difficulties associated with trauma may compromise survivors' abilities to maintain close relationships. Indeed, interpersonal detachment, restricted affect, and heightened irritability are all common PTSD symptoms that can interfere with relational processes known to promote intimacy and broader relationship health (e.g., conflict resolution, provision of socioemotional support). These symptoms in one partner can trigger related responses in the other, which could then function to increase relationship discord—thus facilitating a negative feedback loop that maintains PTSD and relationship dysfunction at both the couple and individual levels. Shared experiences of trauma may also be a source of resiliency for some couples. For example, couples who experienced mutual trauma related to the birthing experience (e.g., miscarriage) may be able to support one another and co-manage related traumatic stress, thus preserving or even enhancing relationship health. Even dyads wherein couples have experienced different traumas may benefit from shared understanding of trauma's psychological impact. Alternatively, others may find their partner's PTSD symptoms overwhelming due to their own unresolved trauma and behave in ways that increase conflict (e.g., make unreasonable demands of partner, engage in the silent treatment when emotionally provoked). These are merely some of the clinical contexts in which PTSD symptoms within couples may be concordant versus discordant; clinicians working with couples experiencing posttraumatic psychopathology may find exploring symptom concordance or discordance in their clients helpful to case conceptualization and, ultimately, intervention. Moreover, given the marked clinical diversity of posttraumatic psychopathology [46], it may be that partners' differential endorsements of certain PTSD symptom clusters (i.e., reexperiencing, avoidance, negative alterations in mood/cognition, and reactivity/arousal) influence relationship functioning. This remains an important area for future research.

We believe our findings have clinical implications. Co-occurrence of PTSD symptoms and relationship distress may be especially pertinent for postpartum populations, for whom the prevalence of mental health conditions is high, and the perinatal period—a time during which mothers-to-be have frequent contact with the healthcare system—may be a window of opportunity during which to engage couples in intervention, particularly those who might not otherwise seek or access care. Moreover, the discrete typologies of couple-level PTSD identified here may be helpful heuristics in clinical settings, as assessing and attuning to trauma history and potential PTSD in expecting parents during the perinatal period could inform treatment recommendations. For example, most PTSD interventions—such as Prolonged Exposure and Cognitive Processing Therapy—are delivered at the individual level, though some clinicians do involve partners occasionally throughout care. However, our results compel systematic consideration of dyadic approaches to PTSD treatment. One notable exception to the individual-level approach to treatment is Cognitive Behavioral Conjoint Therapy for PTSD [47], which is delivered to couples where one partner is diagnosed with PTSD; to our knowledge, no research has yet assessed the intervention among dual trauma couples where one or both partners are experiencing posttraumatic psychopathology. Given the role couple-level conceptualizations of traumatic stress can play in relationship functioning, it may be warranted to examine the efficacy of this and other dyadic interventions in these couples.

To our knowledge, this is the first study to examine PTSD symptoms in couples in a dyadic LPA, the findings from which were largely—though not entirely—consistent with a theory-driven approach. Adopting these two approaches in tandem illuminated the value of considering multiple analytic perspectives. Another methodological strength is the multi-modal, multi-informant assessment of relationship functioning, as self-report measures may potentially inflate associations due to shared method variance. Indeed, couples in this study often scored high on both self-reported PTSD- and relationship-related distress measures, a common problem in the psychopathology in couple literature [48]; utilizing an objective measure of relationship stress increases confidence in findings. Additionally, most research on perinatal psychopathology has examined more advantaged populations, such as White, highly educated, middle-to-upper-middle class women; less is known about how perinatal mental health may function in more sociodemographically diverse samples, including fathers—who are often underrepresented in research on postpartum mental health and functioning [49].

This study has limitations. Data on additional variables of interest—including relationship length, as well as a systematic assessment of trauma-related factors (e.g., lifetime trauma exposure, exposure timing)—were not available. By virtue of when the PTSD and relationship assessments were administered in CCHN, the present design is cross-sectional and precludes directional conclusions. Moreover, the couples in this study were different-gender dyads who had recently given birth to a new baby; these couples may differ from other types of partnerships (e.g., married without kids, same-sex partners). Future relationship science research should strive to study these processes in more diverse samples. It is also unknown whether all individuals were truly trauma-exposed, though sensitivity analyses revealed associations between past-year trauma burden and total PTSD symptom severity in both mothers and fathers. That said, as the PTSD measure was not anchored to an index event, it may be that the symptom assessment is capturing general distress.

## 5. Conclusions

Using data- and hypothesis-driven approaches in a large, diverse sample of mother-father couple dyads, distinct couple subgroups based on both partners' PTSD symptoms—including couples characterized by symptom concordance (*both low*) and discordance (*mother low-father high*, *mother high-father low*)—were elucidated during the postpartum period. These couple-level manifestations of PTSD symptoms were relevant to both maternal and paternal relationship functioning, with partners in couples experiencing elevated PTSD symptoms generally demonstrating more adverse relationship health. Considering couple-level posttraumatic stress may inform more targeted interventions for improving relationships during this transitory time.

## Figures and Tables

**Table 1:** Descriptive statistics for overall sample (*N* = 867 couples).

	Mothers	Fathers	Within-couple correlation
*Individual-level characteristics*			
Age	26.39 (5.70)	29.23 (7.10)	*r* = .728^∗∗∗^
Race/ethnicity			
White	30.2 (262)	26.8 (232)	
Black	44.5 (386)	46.4 (402)	
Latinx	25.3 (219)	24.0 (208)	
Other	—	2.9 (25)	
Education			
< HS graduate	16.3 (141)	21.0 (182)	
HS graduate	38.8 (336)	39.9 (346)	
Some college	24.8 (215)	14.9 (129)	
College graduate or more	19.6 (170)	16.8 (146)	
Other/no information	0.6 (5)	7.4 (64)	
Poverty			
≤100% FPL	38.8 (336)	30.9 (257)	
100-200% FPL	27.1 (235)	21.4 (178)	
>200% FPL	34.1 (296)	47.8 (398)	
First baby	44.5 (386)	—	
Past-year trauma burden	0.82 (0.96)	1.07 (1.20)	*r* = .243^∗∗∗^
Total PTSD symptom severity	26.02 (9.79)	25.69 (9.93)	*r* = .253^∗∗∗^
Observed range	17-79	17-72	
Relationship quality	120.59 (16.57)	122.81 (15.45)	*r* = .558^∗∗^
Observed range	48-151	47-152	
Relationship stress	1.76 (0.82)	1.72 (0.76)	*r* = .681^∗∗^
Observed range	1-5	1-5	
*Couple-level characteristics*			
Average age	27.83 (5.96)		
Minority racial and ethnic couple	67.8 (588)		
Committed relationship	75.5 (655)		
Highest level of education			
< HS graduate	10.5 (91)		
HS graduate	37.9 (329)		
Some college	28.0 (243)		
College graduate or more	23.4 (203)		
Highest level of income			
≤100% FPL	23.4 (203)		
100-200% FPL	25.1 (218)		
>200% FPL	51.4 (446)		

⁣^∗∗∗^*p* < .001, ⁣^∗∗^*p* < .01, and ⁣^∗^*p* < .05.

**Table 2:** Results from dyadic latent profile analyses.

LPA model	Class sizes	Entropy	Log likelihood	AIC	BIC	Adjusted BIC	LMRT, *p* value	BLRT, *p* value
1-class	867		-6426.990	12861.980	12881.040	12868.337	—	—
2-class	772, 95	.929	-6231.778	12477.555	12510.911	12488.680	372.091, *p* < .001	-6426.990, *p* < .0001
3-class	706, 61, 100	.932	-6111.063	12242.127	12289.777	12258.020	230.091, *p* = .005	-6231.778, *p* < .0001
4-class	11, 100, 691, 65	.942	-6050.330	12126.661	12188.606	12147.322	115.762, *p* = .027	-6111.063, *p* < .001
5-class	626, 33, 77, 102, 29	.920	-5997.766	12027.531	12103.772	12052.960	100.193, *p* = .194	-6050.330, *p* < .001

**Table 3:** Associations between LPA-derived couple-level PTSD manifestations and relationship functioning.

	Relationship quality			Relationship stress		
	*β*	95% CI	*p*	*β*	95% CI	*p*
*Mothers*	*R* ^2^ = .12			*R* ^2^ = .18		
Profile: mother high-father low	-0.70	(-1.00, -0.40)	**<.001**	0.77	(0.52, 1.03)	**<.001**
Profile: mother low-father high	-0.61	(-0.96, -0.25)	**.001**	0.71	(0.44, 0.99)	**<.001**
Married and/or cohabitating	0.32	(0.05, 0.59)	**.019**	-0.42	(-0.61, -0.23)	**<.001**
Dyad-level age	0.00	(-0.02, 0.02)	.949	0.00	(-0.02, 0.01)	.870
Dyad-level income						
≤100% FPL	-0.12	(-0.35, 0.12)	.341	0.19	(-0.01, 0.38)	.057
100-200% FPL	0.07	(-0.14, 0.28)	.525	-0.04	(-0.21, 0.12)	.601
>200% FPL	Reference group			Reference group		
Dyad-level education						
< HS graduate	0.30	(0.01, 0.59)	**.043**	0.16	(-0.11, 0.44)	.241
HS graduate	-0.16	(-0.40, 0.07)	.179	0.30	(0.10, 0.49)	**.003**
Some college	-0.12	(-0.35, 0.12)	.332	0.23	(0.04, 0.42)	.016
College graduate or more	Reference group			Reference group		
*Fathers*	*R* ^2^ = .13			*R* ^2^ = .18		
Profile: mother high-father low	-0.57	(-0.84, -0.31)	**<.001**	0.64	(0.39, 0.90)	**<.001**
Profile: mother low-father high	-0.93	(-1.25, -0.60)	**<.001**	0.91	(0.58, 1.23)	**<.001**
Married and/or cohabitating	0.44	(0.19, 0.69)	**.001**	-0.40	(-0.60, -0.20)	**<.001**
Dyad-level age	0.01	(-0.01, 0.02)	.368	0.00	(-0.01, 0.01)	.915
Dyad-level income						
≤100% FPL	0.04	(-0.18, 0.27)	.704	0.31	(0.11, 0.51)	**.003**
100-200% FPL	0.16	(-0.05, 0.37)	.128	0.04	(-0.13, 0.21)	.649
>200% FPL	Reference group			Reference group		
Dyad-level education						
< HS graduate	0.41	(0.09, 0.74)	**.012**	0.15	(-0.16, 0.46)	.330
HS graduate	0.10	(-0.16, 0.36)	.441	0.16	(-0.04, 0.35)	.119
Some college	0.17	(-0.06, 0.40)	.142	0.17	(-0.03, 0.37)	.103
College graduate or more	Reference group			Reference group		

Note: the reference group is *both low*. Associations significant at *p* < .05 are bolded.

**Table 4:** Associations between *a priori* determined couple-level PTSD manifestations and relationship functioning.

	Relationship quality			Relationship stress		
	*β*	95% CI	*p*	*β*	95% CI	*p*
*Mothers*	*R* ^2^ = .14			*R* ^2^ = .19		
Profile: mother high-father low	-0.70	(-0.94, -0.46)	**<.001**	0.63	(0.43, 0.83)	**<.001**
Profile: mother low-father high	-0.21	(-0.47, 0.06)	.126	0.33	(0.13, 0.54)	**.001**
Profile: both high	-0.71	(-1.06, -0.37)	**<.001**	0.89	(0.61, 1.18)	**<.001**
Married and/or cohabitating	0.34	(0.09, 0.60)	**.009**	-0.43	(-0.62, -0.23)	**<.001**
Dyad-level age	0.00	(-0.02, 0.01)	.597	0.00	(-0.01, 0.02)	.754
Dyad-level income						
≤100% FPL	-0.14	(-0.38, 0.09)	.230	0.19	(-0.01, 0.38)	.056
100-200% FPL	0.08	(-0.13, 0.28)	.468	0.28	(-0.22, 0.11)	.528
>200% FPL	Reference group			Reference group		
Dyad-level education						
< HS graduate	0.27	(-0.03, 0.56)	.075	0.20	(-0.08, 0.47)	.156
HS graduate	-0.15	(-0.39, 0.09)	.209	0.28	(0.08, 0.48)	**.005**
Some college	-0.13	(-0.36, 0.10)	.284	0.23	(0.05, 0.42)	**.013**
College graduate or more	Reference group			Reference group		
*Fathers*	*R* ^2^ = .14			*R* ^2^ = .19		
Profile: mother high-father low	-0.27	(-0.49, -0.04)	**.021**	0.47	(0.27, 0.67)	**<.001**
Profile: mother low-father high	-0.68	(-0.92, -0.43)	**<.001**	0.64	(0.42, 0.87)	**<.001**
Profile: both high	-0.85	(-1.15, -0.56)	**<.001**	0.89	(0.59, 1.20)	**<.001**
Married and/or cohabitating	0.46	(0.22, 0.70)	**<.001**	-0.41	(-0.62, -0.20)	**<.001**
Dyad-level age	0.01	(-0.01, 0.02)	.508	0.00	(-0.01, 0.02)	.755
Dyad-level income						
≤100% FPL	0.00	(-0.22, 0.22)	.996	0.32	(0.12, 0.52)	**.002**
100-200% FPL	0.13	(-0.08, 0.34)	.221	0.04	(-0.13, 0.21)	.623
>200% FPL	Reference group					
Dyad-level education						
< HS graduate	0.43	(0.11, 0.75)	**.009**	0.15	(-0.14, 0.45)	.315
HS graduate	0.14	(-0.12, 0.39)	.305	0.12	(-0.08, 0.32)	.223
Some college	0.18	(-0.05, 0.41)	.118	0.16	(-0.04, 0.36)	.108
College graduate or more	Reference group			Reference group		

Note: the reference group is *both low*. Associations significant at *p* < .05 are bolded.

## Data Availability

Study materials (e.g., data and code) are available from the first author upon reasonable request.

## Supplementary Materials

Supplementary Materials We include three tables presenting additional data as part of Supplementary Materials. We first present results from the data-driven LPAs that considered both maternal and paternal symptoms simultaneously; these analyses yielded five distinct solutions. Because scores across profiles can aid in contextualizing results and selecting a theoretically-consistent and optimal solution, Supplementary Table 1 details the mean total PTSD symptom scores for both mothers and fathers across the profiles within each of the five generated solutions. Next, in Supplementary Table 2, we include findings depicting associations between the LPA-derived categorizations from the optimal profiling result—the three-profile solution—and the two relationship functioning measures using different reference groups. For clarity in the main manuscript, we elected to present results using only *both low*dyads as the reference group; we include this extended table with additional reference groups so that interested readers can compare results across other LPA-derived categorizations (e.g., *mother low-father high* vs. *mother high-father low*). Finally, we include a Supplementary Table 3, where associations between the four *a priori* couple-level groupings and two relationship indicators are presented for different reference groups, similar as above; while we retained only *both low* as the reference in the primary analyses presented in-text for clarity, these findings allow readers to review whether other subgroups meaningfully different from one another (e.g., *mother low-father high* vs. *both high*).
