# Supplementary material for: Couple-Level Manifestations of Posttraumatic Stress and Maternal and Paternal Postpartum Relationship Functioning
Source: Depress Anxiety. 2024 Mar 28;2024:6140465. doi: 10.1155/2024/6140465 (PMC11918526; doi:10.1155/2024/6140465)
Supplement: Supplementary Materials — We include three tables presenting additional data as part of Supplementary Materials. We first present results from the data-driven LPAs that considered both maternal and paternal symptoms simultaneously; these analyses yielded five distinct solutions. Because scores across profiles can aid in contextualizing results and selecting a theoretically-consistent and optimal solution, Supplementary Table 1 details the mean total PTSD symptom scores for both mothers and fathers across the profiles within each of the five generated solutions. Next, in Supplementary Table 2, we include findings depicting associations between the LPA-derived categorizations from the optimal profiling result—the three-profile solution—and the two relationship functioning measures using different reference groups. For clarity in the main manuscript, we elected to present results using only both lowdyads as the reference group; we include this extended table with additional reference groups so that interested readers can compare results across other LPA-derived categorizations (e.g., mother low-father high vs. mother high-father low). Finally, we include a Supplementary Table 3, where associations between the four a priori couple-level groupings and two relationship indicators are presented for different reference groups, similar as above; while we retained only both low as the reference in the primary analyses presented in-text for clarity, these findings allow readers to review whether other subgroups meaningfully different from one another (e.g., mother low-father high vs. both high). [file 6140465.f1.docx]

Supplementary Table 1. Maternal and Paternal PTSD Symptoms Across Different Profile Solutions

|  | % (*N*) | Maternal PTSD symptoms  M(SE) | Paternal PTSD symptoms  M(SE) |
| --- | --- | --- | --- |
| **One-profile solution** | | | |
| Class 1 | 100.0 (867) | 26.02 (0.33) | 25.69 (0.34) |
| **Two-profile solution** | | | |
| Class 1 | 88.0 (772) | 25.13 (0.36) | 22.83 (0.31) |
| Class 2 | 12.0 (95) | 32.76 (1.60) | 47.49 (1.82) |
| **Three-profile solution** | | | |
| Class 1 | 81.0 (706) | 22.99 (0.31) | 22.85 (0.28) |
| Class 2 | 7.0 (61) | 26.36 (1.30) | 50.58 (1.89) |
| Class 3 | 12.0 (100) | 47.70 (1.51) | 30.70 (1.63) |
| **Four-profile solution** | | | |
| Class 1 | 1.3 (11) | 53.13 (4.13) | 54.29 (3.78) |
| Class 2 | 11.4 (100) | 45.89 (1.28) | 27.58 (1.05) |
| Class 3 | 79.7 (691) | 22.76 (0.25) | 22.61 (0.31) |
| Class 4 | 7.5 (65) | 25.22 (0.89) | 48.80 (2.30) |
| **Five-profile solution** | | | |
| Class 1 | 72.2 (626) | 22.62 (0.28) | 21.42 (0.23) |
| Class 2 | 3.8 (33) | 25.51 (1.14) | 56.48 (1.70) |
| Class 3 | 8.8 (77) | 46.01 (1.86) | 24.53 (1.29) |
| Class 4 | 11.8 (102) | 25.14 (1.01) | 36.93 (1.12) |
| Class 5 | 3.3 (29) | 32.49 (3.30) | 21.14 (1.88) |

Supplementary Table 2. Associations Between LPA-derived Couple-level PTSD Manifestations and Relationship Functioning, Presented for Different Reference Groups

|  | Relationship quality | | | Relationship stress | | |
| --- | --- | --- | --- | --- | --- | --- |
|  | β | 95% CI | *p* | β | 95% CI | *p* |
| **Reference group: Both low** | | | | | | |
| *Mothers* |  | | |  | | |
| Profile: Mother high—father low | -0.70 | (-1.00, -0.40) | **<.001** | 0.77 | (0.52, 1.03) | **<.001** |
| Profile: Mother low—father high | -0.61 | (-0.96, -0.25) | **.001** | 0.71 | (0.44, 0.99) | **<.001** |
| *Fathers* |  | | |  | | |
| Profile: Mother high—father low | -0.57 | (-0.84, -0.31) | **<.001** | 0.64 | (0.39, 0.90) | **<.001** |
| Profile: Mother low—father high | -0.93 | (-1.25, -0.60) | **<.001** | 0.91 | (0.58, 1.23) | **<.001** |
| **Reference group: Mother high—father low** | | | | | | |
| *Mothers* |  |  |  |  |  |  |
| Profile: Mother low—father high | 0.10 | (-0.36, 0.47) | .678 | -0.06 | (-0.44, 0.32) | .764 |
| *Fathers* |  |  |  |  |  |  |
| Profile: Mother low—father high | -0.35 | (-0.75, 0.02) | .082 | 0.27 | (-0.15, 0.68) | .160 |

*Note:* Models are adjusted for dyad-level relationship commitment, age, poverty, and education.

Supplementary Table 3. Associations Between *A Priori*-determined Couple-level PTSD Manifestations and Relationship Functioning, Presented for Different Reference Groups

|  | Relationship quality | | | Relationship stress | | |
| --- | --- | --- | --- | --- | --- | --- |
|  | β | 95% CI | *p* | β | 95% CI | *p* |
| **Reference group: Both low** | | | | | | |
| *Mothers* |  | | |  | | |
| Profile: Mother high—father low | -0.70 | (-0.94, -0.46) | **<.001** | 0.63 | (0.43, 0.83) | **<.001** |
| Profile: Mother low—father high | -0.21 | (-0.47, 0.06) | .126 | 0.33 | (0.13, 0.54) | **.001** |
| Profile: Both high | -0.71 | (-1.06, -0.37) | **<.001** | 0.89 | (0.61, 1.18) | **<.001** |
| *Fathers* |  | | |  | | |
| Profile: Mother high—father low | -0.27 | (-0.49, -0.04) | **.021** | 0.47 | (0.27, 0.67) | **<.001** |
| Profile: Mother low—father high | -0.68 | (-0.92, -0.43) | **<.001** | 0.64 | (0.42, 0.87) | **<.001** |
| Profile: Both high | -0.85 | (-1.15, -0.56) | **<.001** | 0.89 | (0.59, 1.20) | **<.001** |
| **Reference group: Both high** |  |  |  |  |  |  |
| *Mothers* |  |  |  |  |  |  |
| Profile: Mother high—father low | 0.02 | (-0.39, 0.42) | .942 | -0.26 | (-0.62, 0.09) | .143 |
| Profile: Mother low—father high | 0.51 | (0.09, 0.93) | **.017** | -0.56 | (-0.90, -0.22) | **.001** |
| *Fathers* |  |  |  |  |  |  |
| Profile: Mother high—father low | 0.59 | (0.25, 0.93) | **.001** | -0.42 | (-0.79, -0.06) | **.023** |
| Profile: Mother low—father high | 0.18 | (-0.18, 0.53) | .332 | -0.25 | (-0.63, 0.13) | .196 |
| **Reference group: Mother high—father low** | | | | | | |
| *Mothers* |  |  |  |  |  |  |
| Profile: Mother low—father high | 0.49 | (0.16, 0.83) | **.004** | -0.30 | (-0.57, -0.03) | **.031** |
| *Fathers* |  |  |  |  |  |  |
| Profile: Mother low—father high | -0.41 | (-0.71, -0.11) | **.007** | 0.17 | (-0.12, 0.46) | .245 |

*Note:* Models are adjusted for dyad-level relationship commitment, age, poverty, and education.
